# The Core Subunit of A Chromatin-Remodeling Complex, ZmCHB101, Plays Essential Roles in Maize Growth and Development

**DOI:** 10.1038/srep38504

**Published:** 2016-12-05

**Authors:** Xiaoming Yu, Lili Jiang, Rui Wu, Xinchao Meng, Ai Zhang, Ning Li, Qiong Xia, Xin Qi, Jinsong Pang, Zheng-Yi Xu, Bao Liu

**Affiliations:** 1 Key Laboratory of Molecular Epigenetics of the Ministry of Education (MOE), Northeast Normal University, Changchun 130024, P. R. China; 2 School of Bioengineering, Jilin College of Agricultural Science & Technology, Jilin 132301, P. R. China; 3 Department of Agronomy, Jilin Agricultural University, Changchun 130118, P. R. China

## Abstract

ATP-dependent chromatin remodeling complexes play essential roles in the regulation of diverse biological processes by formulating a DNA template that is accessible to the general transcription apparatus. Although the function of chromatin remodelers in plant development has been studied in *A. thaliana*, how it affects growth and development of major crops (e.g., maize) remains uninvestigated. Combining genetic, genomic and bioinformatic analyses, we show here that the maize core subunit of chromatin remodeling complex, ZmCHB101, plays essential roles in growth and development of maize at both vegetative and reproductive stages. Independent *ZmCHB101* RNA interference plant lines displayed abaxially curling leaf phenotype due to increase of bulliform cell numbers, and showed impaired development of tassel and cob. RNA-seq-based transcriptome profiling revealed that ZmCHB101 dictated transcriptional reprogramming of a significant set of genes involved in plant development, photosynthesis, metabolic regulation, stress response and gene expressional regulation. Intriguingly, we found that ZmCHB101 was required for maintaining normal nucleosome density and 45 S rDNA compaction. Our findings suggest that the SWI3 protein, ZmCHB101, plays pivotal roles in maize normal growth and development via regulation of chromatin structure.

Chromatin-remodeling complexes (CRCs) alter DNA-histone contacts in an ATP-dependent manner, providing essential links between signal transduction and chromatin-based regulation of gene transcription, DNA recombination repair and replication^1^,^2^,^3^,^4^,^5^. SWI/SNF complexes are large, multi-subunit complexes containing eight or more proteins^5^,^6^,^7^. Depending on their types of SUCROSE NONFERMENTING2 (SNF2) family ATPase subunits, the ATP-dependent CRCs are divided into SWITCH2 (SWI2)/SNF2, IMITATION SWITCH (ISWI), Mi-2/Chromodomain-Helicase-DNA binding protein (Mi-2/CHD), and INO80 subfamilies^5^,^6^,^8^. The CRC has a central Snf2-type ATPase which is associated with several core subunits that correspond to orthologs of SNF5, SWI3 and SWP73 in yeast (*Saccharomyces cerevisiae*). In yeast, deletion of genes encoding SWI/SNF subunits causes defects in mating-type switch, sucrose fermentation and transcriptional regulation^9^,^10^. In addition, mutations in mammalian core components of CRCs cause tumorigenesis in somatic tissues of mice and humans, pointing to their essential roles in tumor suppression^4^.

In the model plant *Arabidopsis thaliana*, the roles of SWI/SNF chromatin remodeling in growth and development have been reported^5^,^11^,^12^,^13^,^14^,^15^,^16^,^17^,^18^,^19^,^20^,^21^,^22^. There are four SNF2 ATPases and four SWI3-type proteins in the *A. thaliana* genome. It has been reported that mutations affecting the SWI/SNF subunits caused pleiotropic abnormalities in *A. thaliana* development and responses to phytohormone treatments and environmental stresses^23^. For example, mutations in either *AtSWI3A* or *AtSWI3B*c aused arrest of embryo development at the globular stage, and *AtSWI3B* mutations resulted in death of macrospores and microspores. Moreover, *atswi3c* mutant displayed semi-dwarf stature, inhibition of root elongation, leaf curling, aberrant stamen development and reduced fertility phenotypes. Further, mutations in *AtSWI3D* led to severe dwarfism and alterations in the number and development of flower organs^5^. Thus far, issues regarding how the core subunit of chromatin remodeling complex orchestrates global gene expression in major crops remains unknown.

In this study, using genetic, genomic and bioinformatic analyses, we show that the maize SWI3, ZmCHB101, plays an essential role in maize growth and development. Transgenic lines expressing *ZmCHB101* RNA interference (RNAi) constructs showed markedly altered phenotypes, including abaxially curling leaves, impaired tassel and cob development. Further genome-wide transcriptomic analyses revealed that ZmCHB101 orchestrated the expression of a large set of genes involving metabolic process regulation, photosynthesis, transcriptional regulation and stress response. Intriguingly, we found that ZmCHB101 is required for maintaining normal nucleosome density and 45S rDNA compaction. Our results have elucidated multiple functions of a maize SWI3, ZmCHB101, in mediating transcriptional regulation of a large number of genes essential for normal growth and development of maize via chromatin regulation.

## Results

### Identification of SWI3-type Proteins in Maize

**Figure 1:**
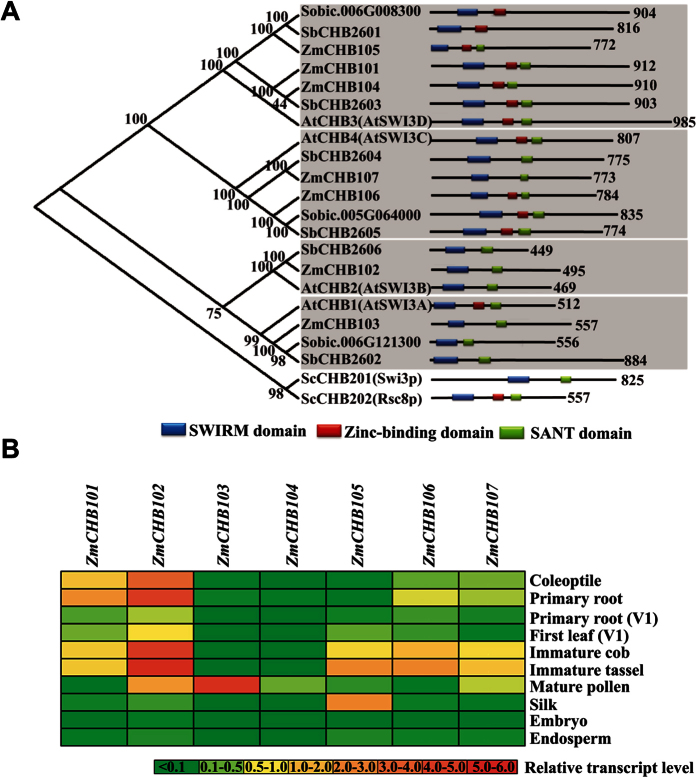
Identification of maize SWI3-type protein-coding genes and examination of their expression patterns in different tissues and conditions. (**A**) Phylogenetic tree of the SWI3-type proteins in plants. The neighbor joining phylogenetic tree constructed by MEGA5 summarizes the evolutionary relationships among different members of the SWI3 from *Arabidopsis thaliana* (At), *Sorghum bicolor* (Sb) and *Zea mays* (Zm). Domains are denoted by colored boxes and intervening regions (including putative domains and unstructured regions) are shown as lines with the total sequence length given at the end. The domains were identified using CDD searching program (http://www.ncbi.nlm.nih.gov/Structure/cdd/wrpsb.cgi). (**B**) Expression patterns of maize *ZmCHBs* in different tissues at different developmental stages. Total RNAs from different tissues at different developmental stages were extracted and used for qRT-PCR analysis. The maize *GAPDH* gene was used as the internal control. Heat color gradation in red and green denote the increase and decrease log_2_-fold change.

To investigate possible functions of the maize SWI3-type proteins in maize growth and development, we first queried the Maize Chromatin Database (http://www.chromdb.org/) and identified four putative genes, which are orthologs of the four *A. thaliana* SWI3 proteins, which were named as ZmCHB101, ZmCHB102, ZmCHB103 and ZmCHB104, respectively. Next, amino acid sequences of the four ZmCHBs were used in independent queries of the Maize Genetics and Genomics Database (http://maizegdb.org), leading to identification of three more maize SWI3 homologs, GRMZM2G139760, GRMZM2G340756 and GRMZM2G119261, which we named as ZmCHB105, ZmCHB106 and ZmCHB107, respectively. Phylogenetic analysis revealed that the seven SWI3 maize proteins could be categorized into four groups: SWI3A, SWI3B, SWI3C and SWI3D, according to their phylogenetic relationships to the *A. thaliana* homologs (Fig. 1A and Table S1)^5^. Specifically, SWI3A (*At2g47620*) includes ZmCHB103, SWI3B (*At2g33610*) includes ZmCHB102, SWI3C (*At1g21700*) includes ZmCHB106 and ZmCHB107, and SWI3D (*At4g34430*) includes ZmCHB101, ZmCHB104 and ZmCHB105. This grouping is consistent with the previous phylogenetic analysis^5^. Notably, there are significantly more SWI3 homologs in maize than in yeast and *A. thaliana*, suggesting the functional diversification of SWI3 paralogs in maize. The characteristic domains in SWI3 including the SWIRM domain (responsible for DNA and nucleosome binding^24^) and the SANT domain (proposed as essential for non-acetylated histone tails^25^,^26^) were identified in all the seven maize SWI3 proteins using the Conserved Domain Database (CDD) searching program (http://www.ncbi.nlm.nih.gov/Structure/cdd/wrpsb.cgi). Furthermore, ZmCHB101, ZmCHB104, ZmCHB105, and ZmCHB106 were found to harbor a zinc-binding domain (Fig. 1A) that could potentially enhance DNA targeting.

To determine the spatial and temporal expression of individual *ZmCHBs*, total RNAs from different tissues including coleoptile, primary root, leaf, cob, tassel, pollen, silk, embryo as well as endosperm were isolated at different developmental stages. Quantitative RT-PCR (qRT-PCR) was performed using gene specific primers (Table S2). We found that *ZmCHB101*, *ZmCHB102*, *ZmCHB106* and *ZmCHB107* were ubiquitously expressed in different vegetative and reproductive tissues such as coleoptile, root, cob as well as tassel (Fig. 1B). Among these, *ZmCHB101* and *ZmCHB102* showed abundant expression in coleoptile, root, cob and tassel. By contrast, expression of *ZmCHB103* could only be detected in mature pollen, while *ZmCHB104* was weakly expressed in mature pollen and could hardly be detected in other tissues. Transcripts of *ZmCHB105* are relatively abundant in reproductive tissues such as cob and silk. Because *ZmCHB101* was generally expressed in both vegetative and reproductive tissues, we chose this gene as a representative to further investigate the potential roles of ZmCHB101.

### ZmCHB101 is Essential for Normal Growth and Development in Maize

**Figure 2:**
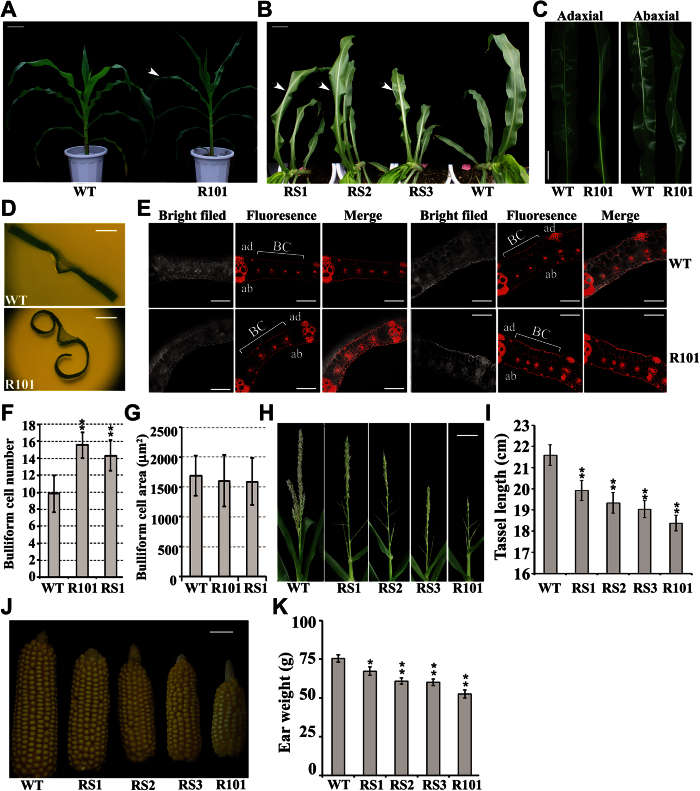
Developmental alterations of *ZmCHB101-RNAi* transgenic plant lines at different stages. (**A**) to (**D**) Developmental alteration caused by *ZmCHB101-RNAi* in leaf tissues. (**A**) and (**B**) Images were taken using V7/V8 stage plants. RS1, RS2, RS3 and R101 represent 4 independent lines of *ZmCHB101-RNAi* plants. Arrows represent leaf-rolling phenotypes. White bars = 10 cm. (**C**) Images were taken using the sixth leaves from V7/V8 stage plants. White bars = 5 cm. (**D**) Cross sections of WT and R101 mature leaf blades. Images were taken using the fourth leaves from 21-day-old plants. White bars = 0.5 cm. (**E**) to (**G**) Cross sections of WT and R101 mature leaf blades showed R101 harbored significantly increased bulliform cell number but same bulliform area compared to WT. (**E**) Cross sections from the 4^th^ mature leaves of WT and R101 plants were stained with propodium iodide (PI) and were examined by a confocal microscope. BC, bulliform cells; ad, Adaxial; ab, Abaxial. White bars = 100 μm. (**F**) Measurement of bulliform cell number quantitatively. **P < 0.01 between *ZmCHB101-RNAi* plants (RS1 and R101) and WT (Student’s *t* test). Error bars indicate SD (n = 10). (**G**) Measurement of bulliform area (μm^2^) quantitatively. Error bars indicate SD (n ≥ 10). (**H**) to (**K**) Developmental alteration caused by ZmCHB101 in reproductive tissues. (**H**) Tassel images were taken at mature stage. RS1, RS2, RS3 and R101 represent 4 independent alleles of *ZmCHB101-RNAi* plants. White bars = 5 cm. (**I**) Measurement of tassel length quantitatively. RS1, RS2, RS3 and R101 represent 4 independent alleles of *ZmCHB101-RNAi* plants. **P < 0.01 between WT and *ZmCHB101-RNAi* lines (RSs and R101) (Student’s *t* test). Error bars indicate SD (n = 20). (**J**) Ear images. RS1, RS2, RS3 and R101 represent 4 independent alleles of *ZmCHB101-RNAi* plants. White bars = 4 cm. (**K**) Measurement of ear weight quantitatively. RS1, RS2, RS3 and R101 represent 4 independent alleles of *ZmCHB101-RNAi* plants. *P < 0.05; **P < 0.01 between WT and *ZmCHB101-RNAi* lines (RSs and R101) (Student’s *t* test). Error bars indicate SD (n = 20).

To investigate the physiological roles of ZmCHB101, we generated transgenic plants harboring *ZmCHB101* RNA interference (RNAi) constructs (RS lines) (Fig. S1A). In parallel, we obtained an independent RNA interference line from the Chromatin Database initiative (http://www.chromdb.org), named R101. Four transgenic progeny RNAi lines (RS1, RS2, RS3 and R101) all showing significant reduction of *ZmCHB101* transcript levels were backcrossed to the B73 as the recurrent parent to minimize potential confounding effects of genetic background (Materials and Methods). The backcross was followed by selfing to obtain homozygous mutant and wild-type (WT) segregates (Fig. S1B). As shown in Fig. S1C and D, the *ZmCHB101* transcripts in different tissues such as leaf, root, tassel and ear were dramatically reduced in all four independent RNAi transgenic lines (RS1, RS2, RS3 and R101), while the expression of *ZmCHB104*, the closest homolog of *ZmCHB101*, was not altered, indicating that *ZmCHB101* transcripts were specifically reduced in these transgenic RNAi lines. We next compared the vegetative development in transgenic RNAi lines and corresponding WT lines. At vegetative stages V6/V7, leaves of all four RNAi lines (RS1, RS2, RS3 and R101) similarly showed a leaf-rolling toward abaxial surface phenotype, and consequently the upper leaves were erected (Fig. 2A–D). To examine the altered phenotypes in more detail, we made cross sections in the 4^th^ mature leaves of R101 and WT. The sections were stained with propodium iodide (PI)^27^ and viewed under confocal microscopy. In sectioned leaves from WT, bulliform cells usually occurred between two vascular bundle ridges in parallel with the more adaxially localized veins and were typically arranged as 8 to 12 cells. In contrast, sections from R101 had significantly more bulliform cells, which is between 14 to 17 cells, regardless of occupying the same area in sections (Fig. 2E–G). This result suggests that ZmCHB101 is involved in bulliform cell division and development.

Reduced expression of *ZmCHB101* also affected reproductive development in the *ZmCHB101-*RNAi lines. Tassels of RNAi lines displayed a sparse appearance due to the reduction of spikelet numbers in comparison to those of WT (Fig. 2H and I). Moreover, ears from *ZmCHB101*-RNAi lines were significantly smaller and consequently had less mass than ears of WT (Fig. 2J and K). Considering the intrinsically higher expression levels of *ZmCHB101* in immature cob and tassel (Fig. 1B), we deduced that ZmCHB101 might play essential roles in reproductive tissue development. Taken together, ZmCHB101 plays crucial roles in both vegetative and reproductive development in maize.

### ZmCHB101 Directs the Landscape of Transcriptional Networks in Maize

**Figure 3:**
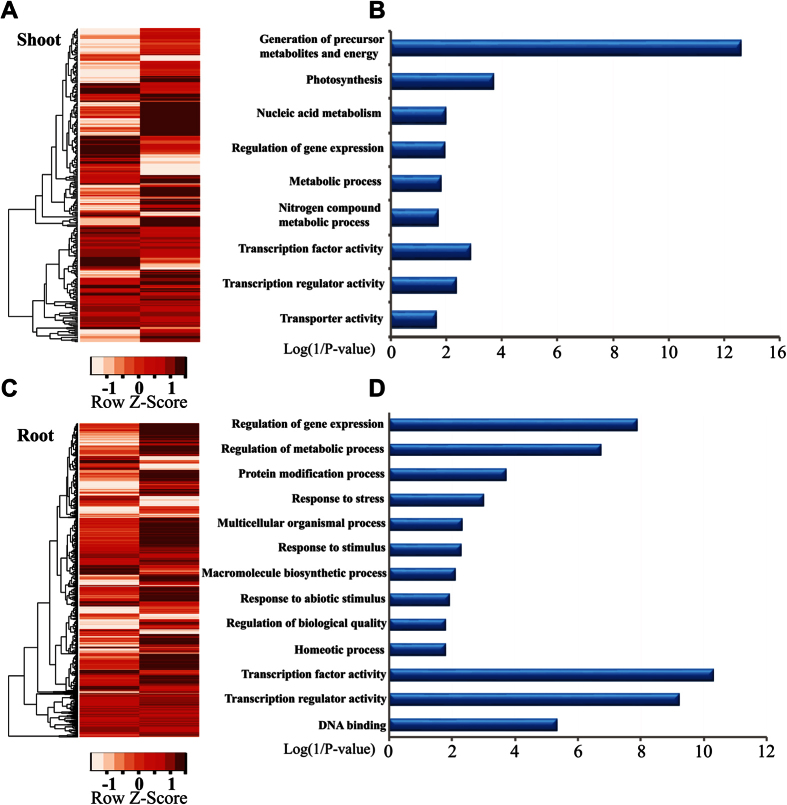
ZmCHB101 regulates expression of a subset of genes in shoot and root of maize seedlings. (**A**) and (**C**) Heatmap of DEGs using hierarchical clustering in shoot and root. DEGs are identified by comparison with wild type (FDR < 0.05). Total RNAs from shoot (**A**) and root (**C**) of 7-day-old WT and R101 maize seedlings were used for mRNA sequencing analysis. Heat color gradation denote log_10_(FPKM). (**B**) and (**D**) The significant enriched biological process and molecular function GO terms of DEGs in shoot (**B**) and root (**D**) are shown (*P*-value < 0.05).

To further explore the potential roles of the putative SWI3 protein, ZmCHB101, we examined how ZmCHB101 governed transcriptional regulation in maize. RNAs from shoot and root tissues were used to construct RNA-seq libraries with two biological replicates. Under stringent statistics and filtering criteria (Materials and Methods), we defined 270 (shoot) and 1315 (root) differentially expressed genes (DEGs) in R101 compared to WT (Table 1 and Table S3). A greater proportion of DEGs were up-regulated in both shoot (157, 58.1%) and root (935, 71.1%) of R101 relative to WT. Strikingly, the transcriptional profile was completely altered in R101 line compared to WT based on hierarchical clustering analysis (Fig. 3A and C), indicating that ZmCHB101 plays essential roles in transcriptional regulation in maize. The target genes of ZmCHB101 were stratified into several distinct biological categories, including metabolic processes, photosynthesis and transcriptional regulation in the shoot (Fig. 3B). A previous study has shown that ectopic expression of *OsACL1* and *OsACL2* induced abaxial leaf curling in rice^28^. Notably, *GRMZM2G047065*, an ortholog of the rice abaxially curled leaf genes (*OsACL1* and *OsACL2*) is significantly induced in R101 (Table 2 and Table S3). In root, ZmCHB101 regulates different metabolic processes, protein modification, stress response, homeostatic process, and transcriptional regulation, which were characterized by GO enrichment analysis (Fig. 3D). Especially, ZmCHB101 regulates expression of genes involved in plant hormone signal transduction process (Table 3), including different receptors, phosphatases and transcriptional regulators in auxin, cytokinin, abscisic acid (ABA), ethylene, jasmonic acid and salicylic acid signaling pathways (Fig. S2 and Table 4), hinting the potential roles of ZmCHB101 in hormone and stress responses. Intriguingly, we noted that ZmCHB101 positively regulates the expression of putative maize PP2Cs but negatively regulates the PYR/PYLs (Fig. S2 and Table 4). PYR/PYL has been shown to act as the ABA receptor in the cytosol, and PP2C is known as a negative regulator in ABA signaling^29^,^30^. This observation prompted us to test whether ZmCHB101 has a role in ABA response. Germinated seeds of two independent ZmCHB101 RNAi lines, R101 and RS1, along with their WT were transferred in 1/2 MS media containing DMSO or 40 μM ABA and dry weight of each line was measured. As shown in Fig. S3A and B, both R101 and RS1 showed a hyposensitive phenotype compared to WT, verifying our hypothesis that ZmCHB101 acts as an important regulator in ABA response. Next, ABA-induced stomata closure was examined in guard cells of epidermal peels of WT, RS1 and R101 lines after being treated with 10 μM ABA for 20 min. As shown in Fig. S3C–F, the stomatal aperture of both R101 and RS1 was larger than that of WT under ABA treatment, indicating that ZmCHB101 positively regulates ABA-mediated stomatal closure. In addition, we found that ZmCHB101 also regulates the expression of a myriad of different transcription factors, such as WRKY, MYB, HB, bHLH, AP2-EREBP (Fig. S4 and Table S4), indicating that ZmCHB101 possibly plays essential roles in transcriptional regulation. To further confirm these results, qRT-PCR was conducted using different marker genes including *GRMZM2G047065*, *GRMZM2G010855*, *GRMZM2G057959*, *GRMZM2G144224*, *GRMZM2G154987* and *AC208201.3_FG002*. As summarized in Table 2, expression patterns of all these marker genes are consistent with the RNA-seq results.

### ZmCHB101 is Required for Maintaining Normal Nucleosome Density and Chromatin Structure

**Figure 4:**
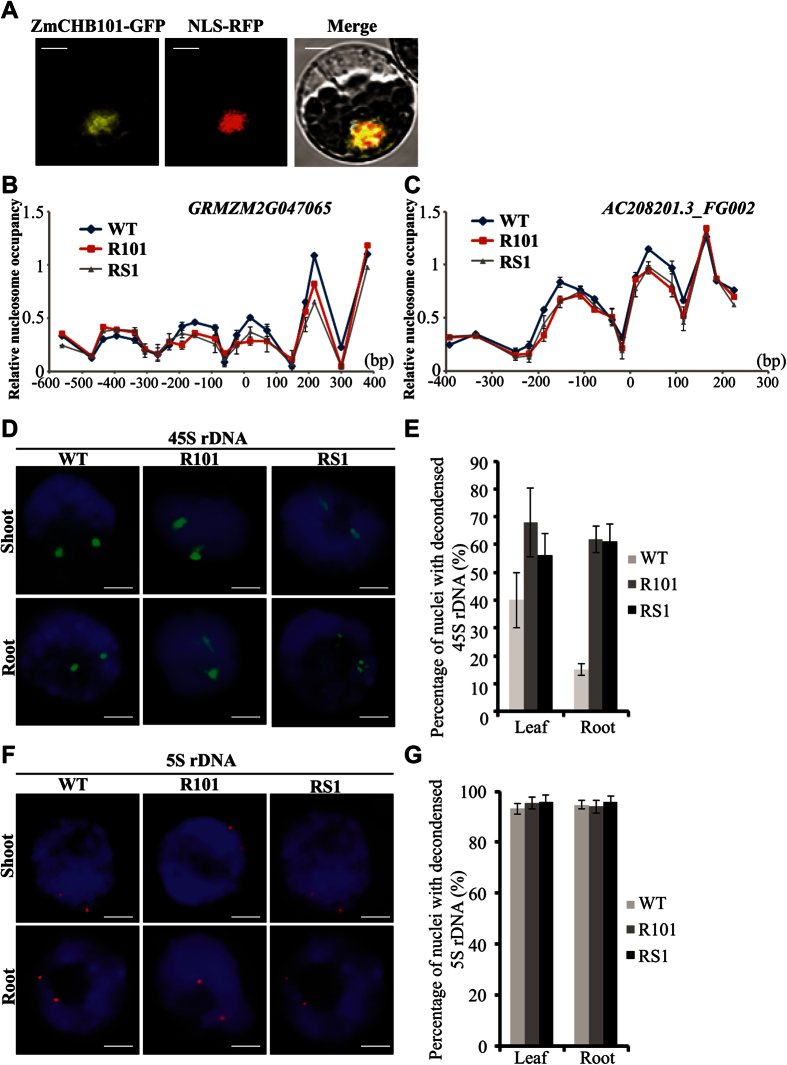
ZmCHB101 is required for maintaining nucleosome density and chromatin structure. (**A**) Subcellular localization of ZmCHB101-GFP. NLS-RFP was included as a marker for labeling the nucleus. GFP, green fluorescent protein; RFP, red fluorescent protein. Bar=20 μm.(**B**) and (**C**) ZmCHB101 is required to maintain high occupancy of the nucleosomes at the upstream and gene body sites at *GRMZM2G047065* (**B**) and *AC208201.3_FG002* (**C**). MNase digestion followed by tiled primer qPCR to monitor nucleosome positioning and occupancy at *GRMZM2G047065* and *AC208201.3_FG002*. MNase qPCR was performed with 7-day-old wild-type (WT), RS1 and R101. The fraction of undigested genomic DNA amplified for each amplicon was used as input control. X-axis denotes distance from the transcription start site and Y-axis denotes relative nucleosome occupancy. Error bars indicate SD (n = 3). (**D**) to (**G**) 45S rDNA chromatin decondensation in R101 and RS1. (**D**) Images of 45S rDNA chromatin decondensation in nuclei from leaves and primary roots of WT, RS1 and R101. Nuclei were subjected to FISH using 45S rDNA probes (green signal). Nuclei were stained with DAPI. Bar = 10 μm. (**E**) Percentage of interphase nuclei with decondensed 45S rDNA fibers in WT, RS1 and R101. 100 nuclei were evaluated in each group. Error bars indicate SD (n = 3). (**F**) Images of 5S rDNA chromatin decondensation in nuclei from leaves and primary roots of WT, RS1 and R101. Nuclei were subjected to FISH using 5S rDNA probes (red signal). Nuclei were stained with DAPI for DNA. Bar = 10 μm. (**G**) Percentage of interphase nuclei with decondensed 5S rDNA fibers in WT, RS1 and R101. 100 nuclei were evaluated in each group. Error bars indicate SD (n = 3).

To further examine the subcellular localization of ZmCHB101, we generated a green fluorescent protein (GFP) reporter construct, *ZmCHB101-GFP*, by inserting GFP downstream of *ZmCHB101*. The *ZmCHB101-GFP* was placed under the control of *CsVMV* (Cassava vein mosaic virus) promoter^31^. Subsequently, *ZmCHB101-GFP* and *NLS-RFP* (used as a marker to label nucleus) were co-transfected into maize protoplasts^31^. As shown in Fig. 4A, GFP signals were confined to the nucleus, indicating that ZmCHB101 localizes primarily to the nucleus.

To investigate the molecular mechanism of how ZmCHB101 regulates the expression of its target genes during development, we examined the nucleosome positioning and occupancy at the *GRMZM2G047065* (Ortholog of *OsACL1*) and *AC208201.3_FG002* (Ortholog of *AtPP2C*) loci, which were identified as markedly differentially expressed genes (Table 2). After performing high-resolution MNase mapping^32^,^33^, we identified well-positioned nucleosomes at the upstream and gene body regions of these loci (Fig. 4B and C). We found that the densities of nucleosomes at multiple sites of these loci were altered in both R101 and RS1 compared with WT (Fig. 4B and C). Specifically, nucleosome densities at the upstream of transcription start sites (UTSs) and gene body regions were dramatically decreased in both RS1 and R101 compared to WT (Fig. 4B and C). To further validate this observation, we performed H3 ChIP-qPCR analysis using anti-H3 antibody in R101 and RS1 along with WT. As shown in Fig. S5 A and B, nucleosome densities at the upstream of transcription start sites (UTSs) were dramatically decreased in R101 and RS1 compared to those of WT, which is consistent with results of the MNase experiment. Especially, the nucleosome density at UTS that is essential for transcriptional regulation was significantly reduced in both R101 and RS1 compared to WT (Fig. S5A and B). It thus appears that decrease of nucleosome densities at UTS due to down-regulation of ZmCHB101 contributes to the higher expression of its target genes in both R101 and RS1 relative to WT. To further test whether ZmCHB101 could directly associate with these loci, we performed ChIP-qPCR analysis using transient expression of ZmCHB101-GFP in maize protoplasts. As shown in Fig. S5C and D, ZmCHB101-GFP but not GFP alone associated with these two loci, strongly suggesting that ZmCHB101 directly associates with these regions and regulate their nucleosome density.

Chromatin assembly and remodeling are believed to be epigenetic switches responsible for the on/off state of rRNA genes^34^,^35^,^36^,^37^. In interphase nuclei, most of 45 S rRNA genes are compacted into heterochromatic chromocenters and the florescent *in situ* hybridization (FISH) signals manifest as compacted spots in WT maize (Fig. 4D). Interestingly, in both R101 and RS1, this well-organized structure was obviously loosened (Fig. 4D and E) compared to WT. We did not observe similar changes at the 5S rDNA region in R101 and RS1 (Fig. 4F and G), indicating chromatin remodeling factor affects 45S rDNA condensation. Next, we performed ChIP-qPCR analysis using anti-H3 antibody to confirm whether nucleosome density at 45S rDNA clusters were altered in the *ZmCHB101-RNAi* lines. As shown in Fig. S5E, the nucleosome densities of different 45S rDNA cluster regions such as promoter region, *18S* and *ITS2* were dramatically decreased compared to WT, indicating that ZmCHB101 is required for maintaining proper nucleosome densities at some loci. Further qRT-PCR analysis revealed that45S rDNA expression were induced in R101 and RS1 compared to WT in both leaf and root (Fig. S5F). To further examine whether ZmCHB101 could directly associate with 45S rDNA cluster, we performed ChIP-qPCR by expressing ZmCHB101-GFP in maize protoplast. As shown in Fig. S5G and H, ZmCHB101-GFP but not GFP could precipitate different sites in 45S rDNA clusters, indicating that ZmCHB101 directly associate with 45S rDNA. To precisely examine the subcellular localization of ZmCHB101, we performed FISH assay with maize protoplasts expressing ZmCHB101-GFP. Intriguingly, GFP signals were found to merge with signals of 45S rDNA probe, suggesting that ZmCHB101-GFP colocalizes with 45S rDNA (Fig. S5I). Taken together, our results using different experimental approaches on independent RNAi lines clearly indicate that ZmCHB101 is directly involved in maintaining proper 45S rDNA condensation.

## Discussion

In the model plant *A. thaliana*, mutations of the genes encoding AtSWI3A or AtSWI3B arrest embryo development at the globular stage. Moreover, mutations of *AtSWI3C* cause leaf curling and reduced fertility, and mutations of *AtSWI3D* lead to leaf curling, severe dwarfism and alteration in the number and development of flower organs and complete male and female sterility^5^. In this work, we studied the function of a major SWI/SNF chromatin remodeling factor in maize. Interestingly, we found that the *ZmCHB101-*RNAi lines also manifested curling leaves and impaired development in reproductive tissues in maize, i.e., significant reduction of spikelet numbers and smaller and lighter ears compared to WT. These observations are reminiscent of the *brahama*, *Atswi3c* and *Atswi3d* mutants in *A. thaliana*^5^. Recently, it was shown that both *A. thaliana* and maize SWI3 associate with ANGUSTIFOLIA3 (AN3), and the association is highly persistent within growing organs in dicots and monocots^38^,^39^. This is consistent with our idea that physiological functions of SWI3 are evolutionarily conserved across different photosynthetic plant species. Functional analyses revealed that the *ZmCHB101*-RNAi lines showed hyposensitive phenotypes to exogenous ABA treatments in terms of seedling growth and ABA-mediated stomata closure. These results indicate that ZmCHB101 likely plays an essential role in ABA-mediated immediate stress responses. Further studies on how ZmCHB101 mediates drought and osmotic stresses may provide additional insights regarding the possible roles of this protein in maize tolerance to abiotic stress conditions.

Genome-wide gene expression profiling revealed that ZmCHB101 regulates expression of a large repertoire of genes involved in metabolic processes, protein modification, stress response, homeostatic process, and transcriptional regulation in the shoot tissue. Of note, we identified that ZmCHB101 negatively regulates the expression of *GRMZM2G047065*, an ortholog of *OsACL1* in rice. Since ectopic expression of *OsACL1* showed abaxially curling leaf phenotypes in transgenic rice plants^28^, it is possible that up-regulated expression of *GRMZM2G047065* contributes to the curling leaf phenotypes in the maize ZmCHB101 RNAi lines. In root, the ZmCHB101-regulated genes were stratified into plant hormone signal transduction process, including different receptors, phosphatases and transcriptional regulators in auxin, cytokinin, ABA, ethylene, jasmonic acid and salicylic acid signaling pathways. Previously, it was reported that *A. thaliana* SWI/SNF complex regulates different hormone signaling pathways and their crosstalk in *A. thaliana*^40^. It is thus possible that CRCs-mediated transcriptional regulation is a conserved feature between *A. thaliana* and maize.

Studies in yeast and animals have documented that the major function of SWI/SNF complexes were the control of nucleosome remodeling at gene promoters and enhancers^41^,^42^. Consistent with these previous findings, we found here that nucleosome occupancy of putative promoter region of two marker genes, *GRMZM2G047065* and *AC208201_FG002*, was significantly decrease in the two independent ZmCHB101 RNAi lines, R101 and RS1, compared with WT, leading to their upregulated expression. Several SWI/SNF subunits in *A. thaliana* have also been shown to interact with different signaling and transcriptional machineries^40^. It is therefore possible that ZmCHB101 acts as the co-activator or suppressor of these components to modulate the expression of its target genes during both normal growth/development and under stress conditions. It has been reported that DNA-binding activators, TATA-binding proteins, and possibly even repressors, would require SWI/SNF when their targeting sites are within nucleosomes^41^,^42^. Moreover, SWI/SNF-activator interactions play an important role in conferring the specificity to target gene promoters^41^,^42^. Thus, in the future, it would be of great interest to identify transcriptional machineries that cooperates with ZmCHB101 in maize under a suite of environmental conditions. In all eukaryotes, the rDNA gene exists as tandemly repetitive clusters, which play essential cellular functions, and its coding regions are highly conserved among eukaryotic species. We showed here that chromatin state at 45S rDNA (but not 5S rDNA) was significantly relaxed in the ZmCHB101 RNAi lines relative to WT, suggesting that fragility of 45S rDNA sites might be enhanced as a result of down-regulation of *ZmCHB101*. Furthermore, we showed that ZmCHB101 could directly associate with 45S rDNA locus and *ZmCHB101*-RNAi caused reduced nucleosome densities at 45S rDNA clusters. These results indicate the ZmCHB101 is essential for regulating 45S rDNA status. Since 45S rDNA also plays an essential role for maintaining genome stability^34^, it is possible that compromised functionality of ZmCHB101 might cause both epigenetic (due to chromatin remodeling) and genetic variations, which, if confirmed, might be explored to generate useful genetic diversities for maize improvement.

## Materials and Methods

### Plant growth and phenotypic analyses

Maize seeds were sterilized in 1% sodium hypochlorite for 5 min and washed in deionized water. The seeds were germinated on moist filter paper at 28 °C for 3 days and were transplanted to enriched soil. Homozygous of *ZmCHB101-RNAi* transgenic and WT segregants from BC4-F4 generation were used for subsequent analyses. In each experiment, 20 plants with different genotypes were used to evaluate leaf curling, flowering state and ear related phenotypes. Shoot and primary root as well as immature cob and tassel was collected for RNA extraction and quantitative reverse transcription-polymerase chain reaction (qRT-PCR). For bulliform cell observation, cross sections of the 4^th^ mature leaves were stained with propodium iodide (PI)^27^ and observed using FLUOVIEW FV1000 Laser Scanning Confocal Microscope (Olympus). Bulliform cell areas were calculated using ImageJ1.49 software (http://imagej.nih.gov/ij/).To measure the ABA sensitivity, sterilized seeds (20 seeds in each experiment) were germinated on moist filter paper and then transferred in 1/2 MS media containing DMSO or 40 μM ABA respectively, and the dry weight was measured at indicated time points (3 d, 5 d, 7 d and 9 d). For ABA mediated stomatal closure analysis, the fully expanded leaves of 4-week-old plants harvested at light condition for 2 hours were rapidly transferred into the solution (10 mMKCl, 25 mM MES with pH = 6.15) for 5 minutes. Subsequently, the samples were treated with DMSO and ABA (10 μM) containing solution for 20 minutes. The leaf samples were stained with PI and stomata was observed and photographed using FLUOVIEW FV1000 Laser Scanning Confocal Microscope (Olympus). Stomatal apertures were calculated using ImageJ 1.49.

### Construction of plasmids

*ZmCHB101* cDNA was isolated from a cDNA library by PCR using gene specific primers ZmCHB101-clone-F and ZmCHB101-clone-R (Table S2). For ZmCHB101-GFP, PCR products of *ZmCHB101* were cloned into 326-GFP vector^31^ using *Xba*I and *Bgl*II. In the case of *ZmCHB101-RNAi* construct generation, gene specific region of *ZmCHB101* coding regions was amplified by PCR primer set ZmCHB101-RNAi-F and ZmCHB101-RNAi-R (Table S2). Subsequently, the PCR product was cloned into pB7GWIWG2 vector^43^ using two recombination reaction steps by taking advantages of Gateway Technology (Invitrogen).

### Generation of transgenic plants

Transformation experiment was conducted with HiII immature embryo by using biolistic bombardment. Twelve-day-old immature embryos were bombarded for cotransformation with 5 mg of gold particles coated with 2 μg plasmid DNA. Bombarded callus was selected on phosphinothricin-supplemented medium and transgenic plantlets were regenerated and hardened off in soil^44^. More than 20 independent RNAi induced gene silencing T_0_ transformants (RS lines) were obtained. The R101 RNA interference mutant line was obtained from the Plant Chromatin Database initiative (http://www.chromdb.org/; T-MCG5812.05 locus, Maize Stock Center number 3201-35). Regenerated T_0_ plants were introgressed into the B73 recurrent parent. The crossing scheme adopted to achieve homozygosity for the transgene and wild-type segregant plants used for molecular and phenotypic analysis is described in Supporting Information. The presence of the transgene was validated for bialophos (BASTA) herbicide resistance and with PCR detection. Characterization of changes in RNA level was performed using qRT-PCR analysis with *ZmCHB101* specific primers (Table S2). RNA and DNA used for molecular analyses were extracted from the first mature leaf of seedlings at V2/V3 stage, which were grown in the thermostat incubator with 16 h light at 28 °C and 8h dark at 22 °C.

### Sequence alignment and phylogenetic analysis

To identify SWI3 homologues and orthologs in *Zea mays*, *A. thaliana*, *Sorghum bicolor* and yeast, the amino acid sequences of SWI3 in *A. thaliana* were subjected to Blastp searches against the genomic database in *Zea mays* (http://www.maizegdb.org/), *Sorghum bicolor* (http://www.plantgdb.org/SbGDB/) and the Chromatin Database (http://chromdb.org/). Conserved domain analysis was performed using CDD program (http://www.ncbi.nlm.nih.gov/Structure/cdd/wrpsb.cgi). The phylogenetic trees were constructed using the neighbor-joining tree after align the protein sequences in MEGA5 software^45^, in which the number of bootstrap replication was 1000.

### RNA isolation and qRT-PCR analysis

Total RNAs were isolated using Trizol Reagent (Invitrogen) according to the manufacturer’s protocol. We incubated 2 μg RNA with DNase I (Invitrogen) and prepared first strand cDNA using SuperScript^TM^II reverse transcriptase (Invitrogen) in a total reaction volume of 20 μl. qRT-PCR was carried out using the StepOnePlus^TM^ Real-Time quantitative RT-PCR system (Applied Biosystems) with the TransStart^TM^ Top Green qPCR SuperMix reagent (TransGen Biotech). We mixed each 20 μl cDNA preparation with 120 μl TE buffer (pH = 8.0) and used 0.5 μl of the diluted cDNA as a PCR template. The gene-specific primers were listed in Table S2. The primers were designed using Primer Premier 5.0 (http://www.PremierBiosoft.com). Three independent replicates were carried out for each sample-primer combination and the 2^−△△CT^ method was used to calculate the steady-state mRNA level for each gene or 2^−△CT^ method was used for expression assay of maize ZmCHBs from different tissues at distinct developmental stages. Maize *GAPDH* gene was used as the internal control^46^.

### RNA-seq analyses and data validation

Maize seedlings were grown in enriched soil and total RNA extracted from shoot and root of 7-day-old WT and *ZmCHB101-RNAi* (R101) were used for mRNA sequencing analysis. Two biological replicates were conducted for each sample and sequenced. The libraries of shoots and roots were generated and sequenced by taking advantages of HiSeq2000 (Illumina, USA). Library construction and sequencing analysis were carried out with standard protocols (Illumina, USA). Raw data were cleaned by removing adaptor contamination and low quality reads by Fastx-tools (http://hannonlab.cshl.edu/fastx_toolkit/). For each library, more than 11 million clean reads (Q20 > 90%) were obtained (Table S5). Clean data have been deposited at the SRA database (http://www.ncbi.nlm.nih.gov/sra/) with accession number SRP068071. The clean data were mapped against B73 RefGen_v2 with corresponding annotation by Tophat2.0 (http://tophat.cbcb.umd.edu/) using default parameters. The aligned results were then used to assess the FPKM (Fragments per Kiloblase of transcript per Million mapped reads) and expression differentiation by Cuffdiffv2.0.1. The FDR-adjusted p-value (q value) of the test statistic was used for identify differentially expressed genes. To reduce the influence of transcription noise, a given gene was determined to express when its FPKM value≥1. The genes showing an absolute value of log_2_(FPKMR101/FPKMWT) ≥0.7 and adjusted p value (FDR)<0.05 were considered as differentially expressed genes. To verify RNA-seq results, possible leaf curling- and ABA signaling-related genes including *GRMZM2G047065*, *AC208201.3_FG002*, *GRMZM2G010855*, *GRMZM2G057959*, *GRMZM2G144224* and *GRMZM2G154987* were selected for qRT-PCR analysis. Expression values by qRT-PCR were calculated by relative expression of genes to *ZmACT1 (GRMZM2G126010*)^47^. To investigate the functional relevance of differentially expressed genes of each type between WT and RNAi line, we performed GO enrichment analysis with complete GO assignments from AgriGO (http://bioinfo.cau.edu.cn/agriGO/). All GO terms containing differentially expressed genes in each comparison were tested by hypergeometric test and only GO terms with *P-*value < 0.05 were regarded as significantly enriched. Furthermore, KEGG (Kyoto Encyclopedia of Genes and Genomes, http://www.kegg.jp/) enrichment analysis was also performed using the DEGs to test the biological pathways affected by ZmCHB101. Pathways with *q-*value < 0.05 were regarded as significantly enriched.

### Subcellular localization

Subcellular localization analysis was performed by using maize leaf protoplasts. Protoplasts were prepared from leaf tissues from 15-day-old maize plants grown on soil. The middle parts of the second leaves were cut into 1 mm strips and digested in an enzyme solution (0.6 M mannitol, 10 mM MES, pH 5.7, 1.5% (w/v) cellulose R10 (Yakult Pharmaceutical Ind. Co., Ltd., Japan), 0.5% (w/v) macerozyme R10 (Yakult Pharmaceutical Ind. Co., Ltd., Japan), 1 mM CaCl_2_, 5mM β-mercaptoethanol and 0.1% (w/v) bovine serum albumin) in the dark for 3 h with gentle shaking. The protoplasts were harvested by filtering through a 35 μm nylon mesh and washed once with 0.6 M mannitol, then suspended in a transfection buffer (0.6 M mannitol, 15 mM MgCl_2_, 4 mM MES,pH 5.7) to a concentration of 2 × 10^5^ protoplasts ml^−1^. Plasmid DNAs were prepared using Qiagen columns. Protoplasts were transfected with *ZmCHB101-GFP* and *NLS-RFP*, a chimeric red fluorescent protein (RFP) construct containing a nuclear localization signal. About 20 μg plasmid DNA was mixed with 200 μl protoplasts. Then, 220 μl PEG solution (0.8 M mannitol, 100 mM CaCl_2_, 40% PEG 4000) was immediately mixed with the protoplasts by gently shaking, and then incubated for 15 min at 25 °C. After incubation, the protoplasts were washed with 1 ml W5 solution (154 mM NaCl, 125 mM CaCl_2_, 5 mM KCl, 2 mM MES, pH 5.7) and collected by centrifugation at 70 × *g* for 3 min. The protoplasts were resuspended in W5 and incubated in the dark at 25 °C overnight^48^. GFP or RFP fluorescence signals were observed under Laser Scanning Confocal Microscope (Olympus).

### MNase Assay

A total of 5 g shoot or root from7-d-old plants was harvested in liquid nitrogenafter cross-linking in 1% formaldehyde. Nuclei and chromatin were isolated as previously described^49^ with some changes. The isolated nuclei were washed three times with isolation buffer (10mM Tris-Cl pH 8.0, 0.1 M MgCl_2_, 0.1 M NaCl, 0.1% Triton-X, 10 mM β-mercaptoethanol), and the isolated chromatin was digested with 0.5 units/ml (final concentration) of Micrococcal Nuclease (Takara) for 10 min in digestion buffer at 37 °C. Subsequent steps were performedas previously described^50^. Mononucleosomes were excised from 1.2% agarose gels and purified using a gel purificationkit (Qiagen). The purified DNA was quantified using a NanoDrop ND-2000 spectrophotometer. 20 ng of purified DNA were used for qPCR to monitor nucleosome occupancy. The fraction of input was calculatedas 2^−[Ct(mono)−Ct(gDNA)]^ using undigested genomic DNA. The tiled primer sets used for realtime PCR are listed in Table S2.

### ChIP-qPCR analysis

ChIP-qPCR assay for testing nucleosome density was performed as described previously^34^ with slight modifications. Chromatin was isolated and sheared to 200–800 bp with M220 Focused-ultrasonicator (Covaris). Soluble chromatin was incubated with anti-H3 antibody (Abcom, ab1791) or rabbit serum overnight at 4 °C. DNA was recovered by phenol/chloroform extraction and ethanol precipitation.ChIP qPCR for ZmCHB101 binding assay were performed with maize leaf protoplasts. Protoplasts were transfected with *ZmCHB101-GFP*or *GFP*and 5 × 10^6^ protoplasts were subjected to ChIP analysis for each sample. ChIP assays were performed using EpiTect ChIP OneDay Kit (Qiagen,#334471) according to the manufacturer’s protocol. Soluble chromatin was incubated withanti-FLAG antibody (Sigma, F3165) or rabbit serum overnight at 4 °C. Following ChIP, quantitative realtime PCR were performed with 45S rDNA sepecific primers^34^ or promoter regoins of *GRMZM2G047065* and *AC208201_FG002* (Table S2).

### Fluorescence *in situ* hybridization (FISH) assay

Nuclei isolation were performed using the procedure described by Huang*et al*.with minor modifications^34^. An appropriate amount of leaves or roots were chopped innucleiextraction buffer (0.01 M MgSO_4_, 5 mM KCl, 0.5 mM Hepes,1 mg/ml dithiothreitol, and 0.25% Triton X-100, pH 7.0) andfiltered with Miracloth (Merck Millipore). The nuclei were centrifuged at 200 g for 10 min at 4 °C and resuspended in thesame buffer. Then, nuclei were fixed in 4% paraformaldehyde in1×PBS for 1 h at the room temperature and spread on slides.The protocol for FISHwere essentially as described in Zhang*et al*.^51^. 45S rDNA and 5S rDNA repetitive DNA sequences were labeled with Alexa Fluor 488-5-dUTP (green coloration) and Texas Red-5-dCTP (red coloration), respectively, and hybridized to the slides. Slide denaturation, hybridization, and washing conditions were carried out following the manufacturer’s recommendations (Invitrogen; no. C11397). Slides were examined with an Olympus BX61 fluorescence microscope and digitally photographed. To examine colocalization of ZmCHB101-GFP and 45S rDNA, 45S rDNA was labeled with Texas Red-5-dCTP (red coloration) in nulears extracted from maize protoplast transfected with *ZmCHB101-GFP*. Detection of 45S rDNA and GFP fluorescence signals was performed as descrbied.

## Additional Information

**How to cite this article**: Yu, X. *et al*. The Core Subunit of A Chromatin-Remodeling Complex, ZmCHB101, Plays Essential Roles in Maize Growth and Development. *Sci. Rep.* **6**, 38504; doi: 10.1038/srep38504 (2016).

**Publisher's note:** Springer Nature remains neutral with regard to jurisdictional claims in published maps and institutional affiliations.

## Supplementary Material

Supplementary Information

Supplementary Tables

## Figures and Tables

**Table 1:** Summary of differentially expressed genes (DEGs) in shoot and root of maize seedlings from R101 compared to those from WT revealed by RNA-seq.

Tissue	Total No. (%) of DEGs	No. (%) of up-regulated genes	No. (%) of down-regulated genes	Total No. of expressed genes
Shoot	270 (0.82)	157 (58.1)	113 (41.9)	33095
Root	1315 (3.70)	935(71.1)	380 (28.9)	35561

**Table 2:** Real-time quantitative RT-PCR confirmation of selected DEGs.

Gene ID	WT	R101	qRT-PCR*				Arabidopsis or rice ortholog
	FPKM	FPKM	R101	RS1	RS2	RS3	
GRMZM2G047065	5.4	10.6	2.4	2.9	2.2	1.9	OsACL1 and OsACL2
GRMZM2G010855	4.4	39.1	12.2	8.8	7.6	8.5	AT1G07430.1 (AIP1)
GRMZM2G057959	32.87	12.97	0.36	0.42	0.48	0.76	AT5G05440.1 (PYL5)
GRMZM2G144224	64.46	28.52	0.31	0.55	0.48	0.41	AT5G05440.1 (PYL5)
GRMZM2G154987	14.16	6.19	0.33	0.67	0.32	0.37	AT2G26040.1 (PYL2)
AC208201.3_FG002	27.51	118.40	5.5	4.0	2.7	3.6	AT2G30020.1 (PP2C)

*Total RNAs were isolated from shoot and root for GRMZM2G047065 and GRMZM2G010855, GRMZM2G057959, GRMZM2G144224. GRMZM2G154987 and *AC208201.3_FG002*, respectively, and subjected to qRT-PCR analysis. Fold change of gene expression in RNAi lines relative to WT were presented. *ZmACT1* was used as an internal control.

**Table 3:** KEGG enrichment analysis for DEGs in shoot and root of R101.

Tissue	KEGG Pathway	KEGG ID	No. of DEGs	Total genes	Correct *P* value
shoot	Photosynthesis	zma00195	10	112	5.48E-07
	Oxidative phosphorylation	zma00190	10	180	2.23E-05
	Metabolic pathways	zma01100	26	1584	0.004
	Flavonoid biosynthesis	zma00941	2	17	0.020
root	Plant-pathogen interaction	zma04626	27	131	0
	Plant hormone signal transduction	zma04075	24	186	9.83E-10
	Zeatin biosynthesis	zma00908	7	18	2.14E-06

**Table 4:** FPKM of DEGs involved in Plant hormone signal transduction pathway in roots between wild type and R101.

Pathway	Related hormone signal	Gene family	Gene ID	FPKM		Description of gene family
				WT	R101	
Plant hormone signal transduction (zma04075)	ABA	PYR/PYL	GRMZM2G057959	32.87	12.97	abscisic acid receptor PYR/PYL family
			GRMZM2G144224	64.46	28.52	
			GRMZM2G154987	14.16	6.19	
		PP2C	GRMZM2G010855	4.4	39.15	protein phosphatase 2C
	Auxin	SAUR	GRMZM2G479596	46.16	15.72	SAUR family protein
			GRMZM2G430052	60.55	19.57	
			GRMZM2G330012	46.12	18.23	
			GRMZM2G154332	21.39	7.71	
	Cytokinine	A-ARR	GRMZM2G040736	20.63	38.85	two-component response regulator ARR-A family
			GRMZM2G129954	57.79	23.28	
	Ethylene	EIN3	GRMZM2G151811	1.15	3.11	ethylene-insensitive protein 3
	Jasmonic acid	COI1	GRMZM2G353209	15.24	8.89	coronatine-insensitive protein 1
		JAZ	GRMZM5G838098	9.48	46.61	jasmonate ZIM domain-containing protein
			GRMZM2G145412	14.55	122.49	
			GRMZM2G145458	10.95	57.08	
			GRMZM2G089736	109.62	288.24	
			GRMZM2G005954	4.22	9.55	
			GRMZM2G343157	5.16	27.18	
			GRMZM2G173596	12.38	91.56	
			GRMZM2G101769	48.5	91.79	
			GRMZM2G445634	55.18	210.64	ZIM motif family protein
			GRMZM2G036351	54.25	273.71	
	Salicylic acid	TGA	GRMZM2G445575	69.9	36.48	transcription factor TGA
			GRMZM2G060290	18.95	10.22	
