# Supplementary material for: The Core Subunit of A Chromatin-Remodeling Complex, ZmCHB101, Plays Essential Roles in Maize Growth and Development
Source: Sci Rep. 2016 Dec 5;6:38504. doi: 10.1038/srep38504 (PMC5137073; doi:10.1038/srep38504)
Supplement: Supplementary Information [file srep38504-s1.pdf]

The Core Subunit of A Chromatin-Remodeling Complex, ZmCHB101, Plays Essential Roles in Maize Growth and Development

Xiaoming Yu[1,2,†], Lili Jiang[1,†], Rui Wu[1,† #], Xinchao Meng[1], Ai Zhang[1], Ning Li[1], Qiong Xia[1], Xin Qi[3], Jinsong Pang[1], Zheng-Yi Xu[1,*], Bao Liu[1,*]

[1]Key Laboratory of Molecular Epigenetics of the Ministry of Education (MOE), Northeast Normal University, Changchun 130024, P. R. China

[2]School of Bioengineering, Jilin College of Agricultural Science & Technology, Jilin 132301, P. R. China

[3]Department of Agronomy, Jilin Agricultural University, Changchun 130118, P. R. China

[#]Current address: Max Planck Institute for Developmental Biology, Department of Molecular Biology, Tuebingen 72076, Germany

[†]These authors contributed equally to this study.

*Correspondence could be addressed to Zheng-Yi Xu (xuzy100@nenu.edu.cn) or Bao Liu (baoliu@nenu.edu.cn)

List of Supplementary Information

Fig. S1. Generation of *ZmCHB101-RNAi* plants.

Fig. S2. Illustration of plant hormome signal transduction process based on KEGG analysis for the differentially expressed genes.

Fig. S3. ZmCHB101 plays essential roles in ABA responses.

Fig. S4. Classification of defferantially expressed TFs in root of R101.

Fig. S5. ZmCHB101 is required for maintaining nucleosome density and associates with *GRMZM2G047065*, *AC208201.3_FG002* and 45S rDNA.

Table S1. Alignment of SWI3 proteins from Yeast, *Arabidopsis*, Sorghum and maize.

Table S2. List of primers used for gene cloning, vector construction and gene expression assay.

Table S3. Expression values of differentially expressed genes in shoot and root between WT and R101.

Table S4. List of differentially expressed TFs in root of R101.

Table S5. RNA-seq data information.

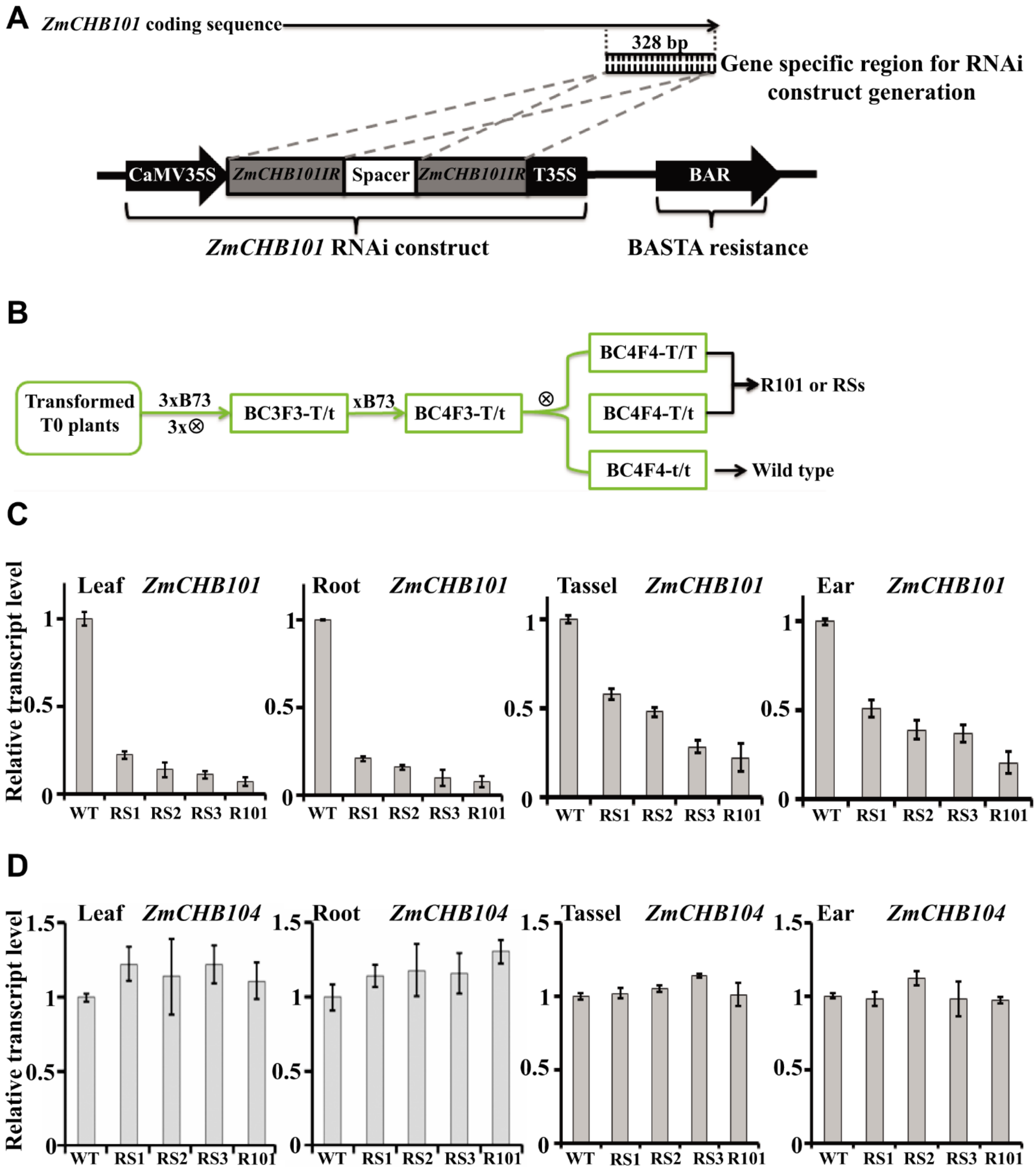

Fig. S1. Generation of *ZmCHB101-RNAi* plants. (**A**) Schematic representation of *ZmCHB101-RNAi* construct. Inverted repeats of *ZmCHB101* specific region (*ZmCHB101IR*) were inserted into pB7GWIWG2 vector flanked by a spacer sequence. CaMV35S and T35S: 35S promoter and terminator regions of *Cauliflower mosaic virus*, respectively; Spacer: the second intron of *AT5G27430*. (**B**) Crossing scheme for

ZmCHB101-RNAi plants. The crossing scheme used in this study is illustrated. After each cross, the presence of the transgene was validated by BASTA resistance and PCR detection. Due to the dominant effect of transgene, the identification of the segregate genotypes requires two sequential selfing generations and a test of transgene segregation was done on the second selfing generation. Specifically, BASTA and PCR screening are conducted on a minimum of 20 phytotron-grown seedlings derived from each selfed ear. Introgression into B73 (3 total outcrosses) was completed only for lines selected for the lowest *ZmCHB101* expression level. Self-crossing of BC4F3 heterozygous (T/t) led to the BC4-F4 generation including WT and *ZmCHB101-RNAi* plants (RS1, RS2, RS3 and R101). These plants were further used for subsequent phenotypic and molecular analyses. (**C**) and (**D**) Examination of *ZmCHB101* transcripts in *ZmCHB101-RNAi* plants in leaf, root, immature tassel and immature ear. Total RNAs were extracted from leaf, root, immature tassel and immature ear of WT and 4 independent ZmCHB101 RNAi plants (RS1, RS2, RS3 as well as R101) and subsequently qRT-PCR analyses were performed. *ZmCHB104* that showed highest similarity of nucleotide sequence to *ZmCHB101* was used as the negative control for specific reduction of *ZmCHB101* transcripts. *ZmACT1* was used as an internal control. Error bars indicate SD (n=3).

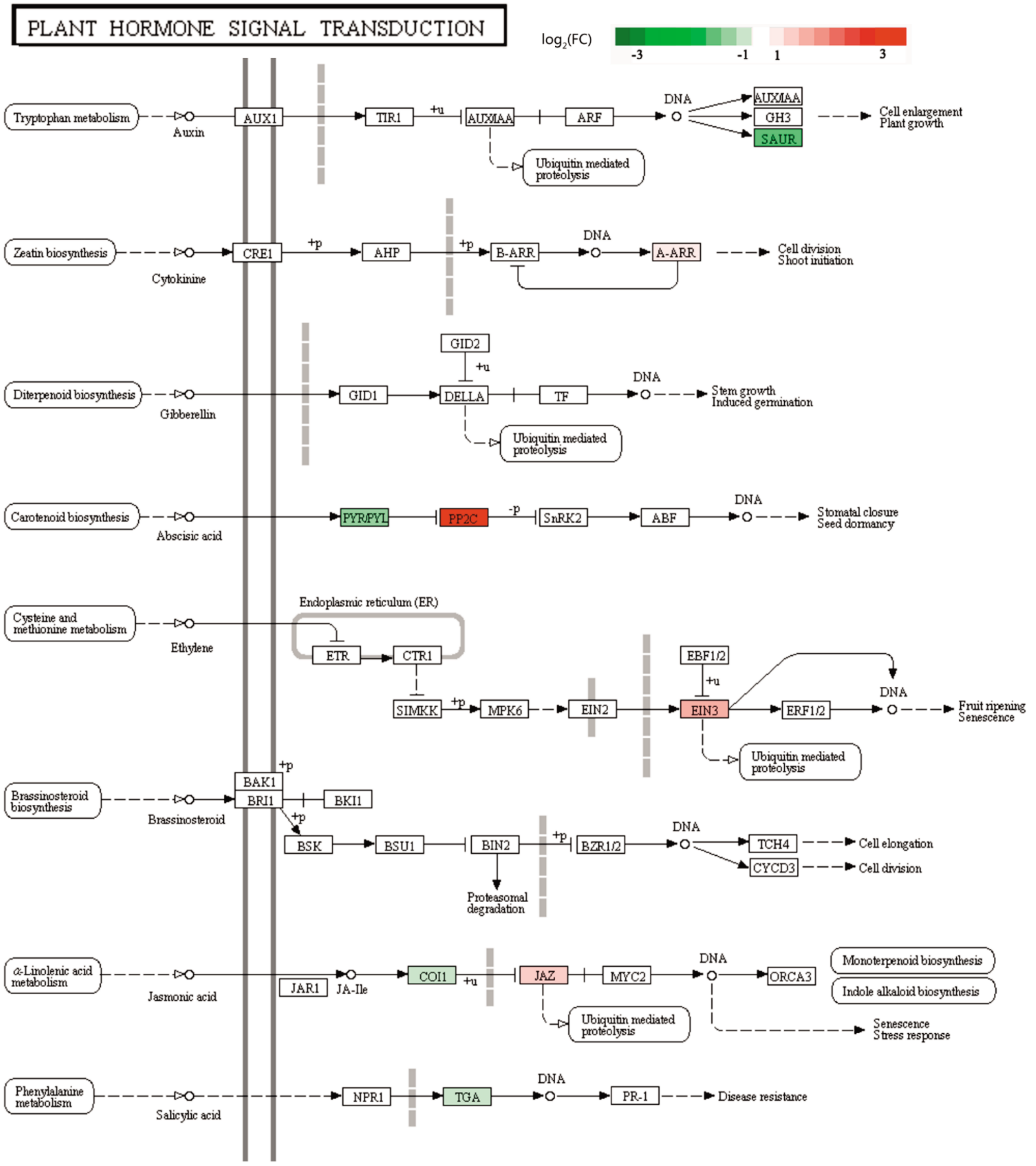

Fig. S2. Illustration of plant hormome signal transduction process based on KEGG analysis for the differentially expressed genes. KAAS (KEGG automatic Annotation Server) provides functional annotation of genes by BLAST comparisons against the KEGG genes database. The result contains automatically generated KEGG pathways. Red box, up regulated genes; green box, down regulated genes.

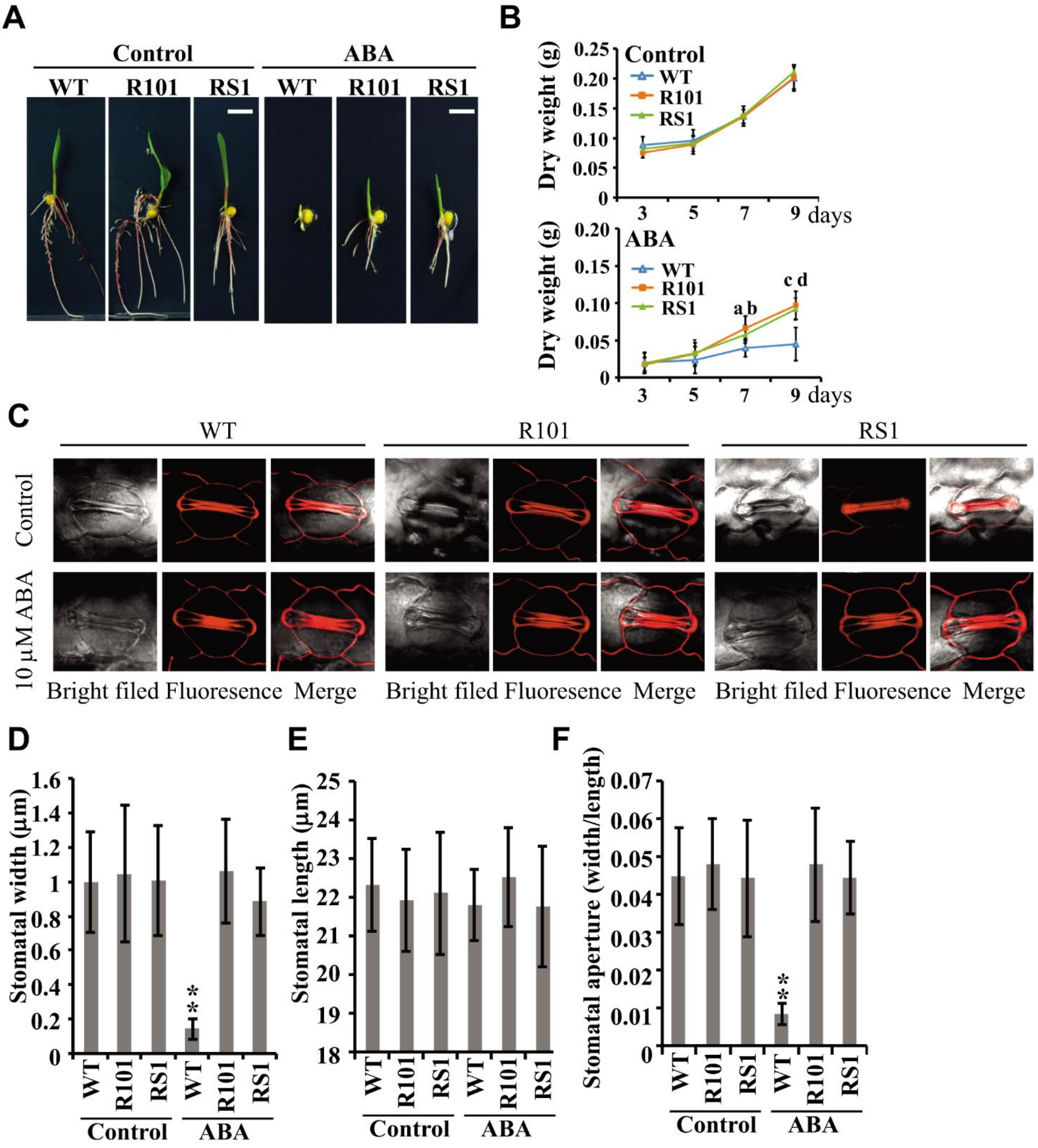

Fig. S3. ZmCHB101 plays essential roles in ABA responses. (**A**) and (**B**) Effect of exogenous ABA on seedling growth. WT, RS1 and R101 plants were planted on media supplemented with DMSO or ABA (40 μM). (A) Images of the indicated plants were taken at 7 days after being transferred. White bars=1cm. (**B**) Dry weights of plants were measured at indicated time points. Statistical analysis was performed between RS1, R101 and WT. Error bars indicate SD (n=10). **P*<0.05; ***P*<0.01 between RS1, R101 and WT (Student's *t* test). (**C**) Maize leaves were treated with 10 μM ABA for 20 min and subsequently were stained with propodium iodide. (**D**) to (**F**) Role of ZmCHB101 in ABA-induced stomata closure. Fully opened stomata of WT, RS1 and R101 were

treated with 10 μM ABA for 20 min and the width (**D**), length (**E**) and the ratio between width/length (**F**) were measured in a triplicate experiment with more than 20 pairs of guard cells per experiment. Error bars indicate SD (n=3). Statistical analysis was performed by comparing RS1, R101 and WT. **$P<0.01$ between RS1, R101 and WT (Student's *t* test).

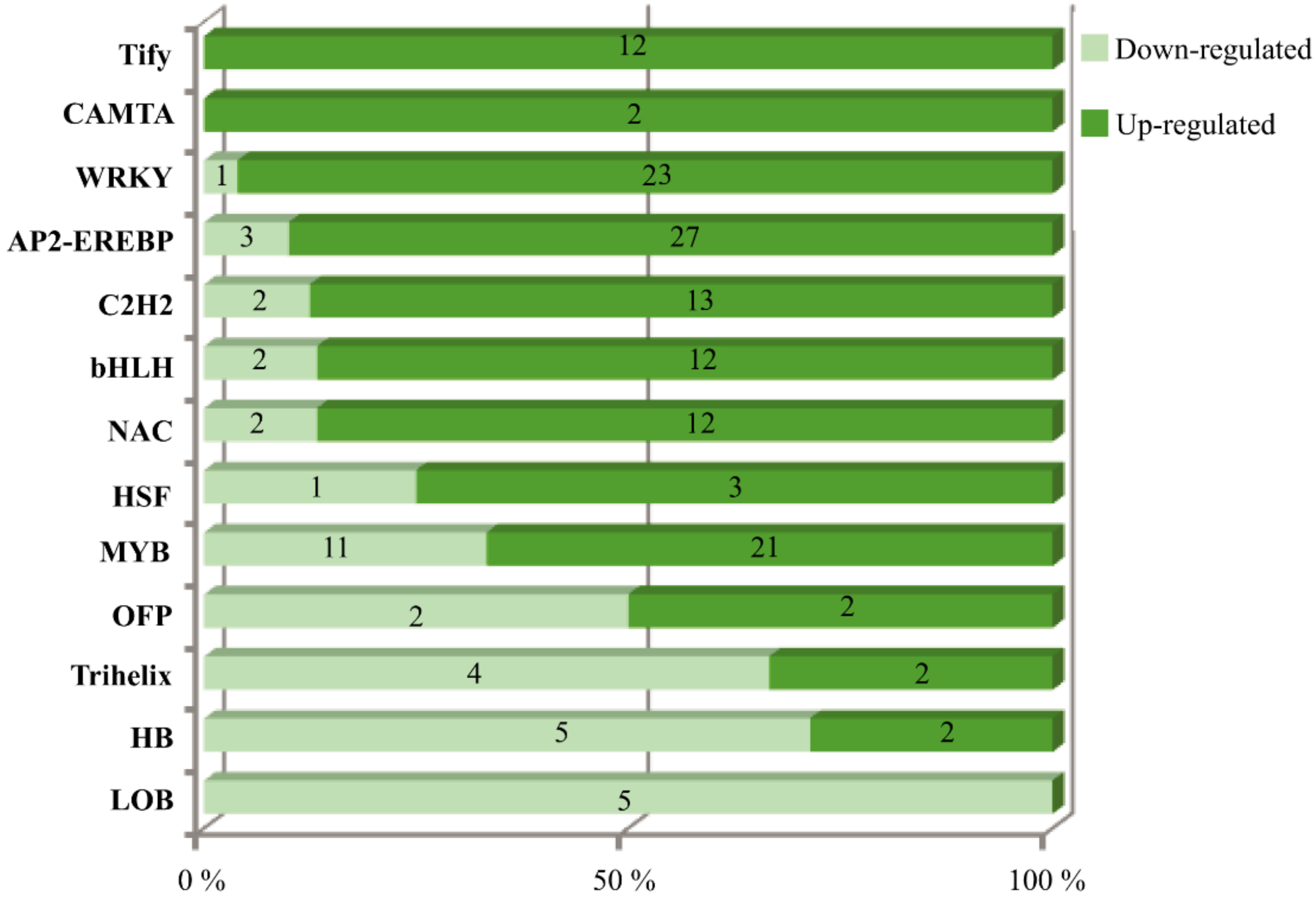

Fig. S4. **Classification of defferantially expressed TFs in root of R101.** 169 out of 1315 root DEGs were classifiedas transcription factors (TFs) by iTAK database 13.07 (http://bioinfo.bti.cornell.edu/cgi-bin/itak/db_home.cgi). These 202 TFs was distributedinto 14 TF classes as indicated. 131 out of these 169 transcription factors (77.5%) were up regulated and 38 (22.5%) were down regulated.

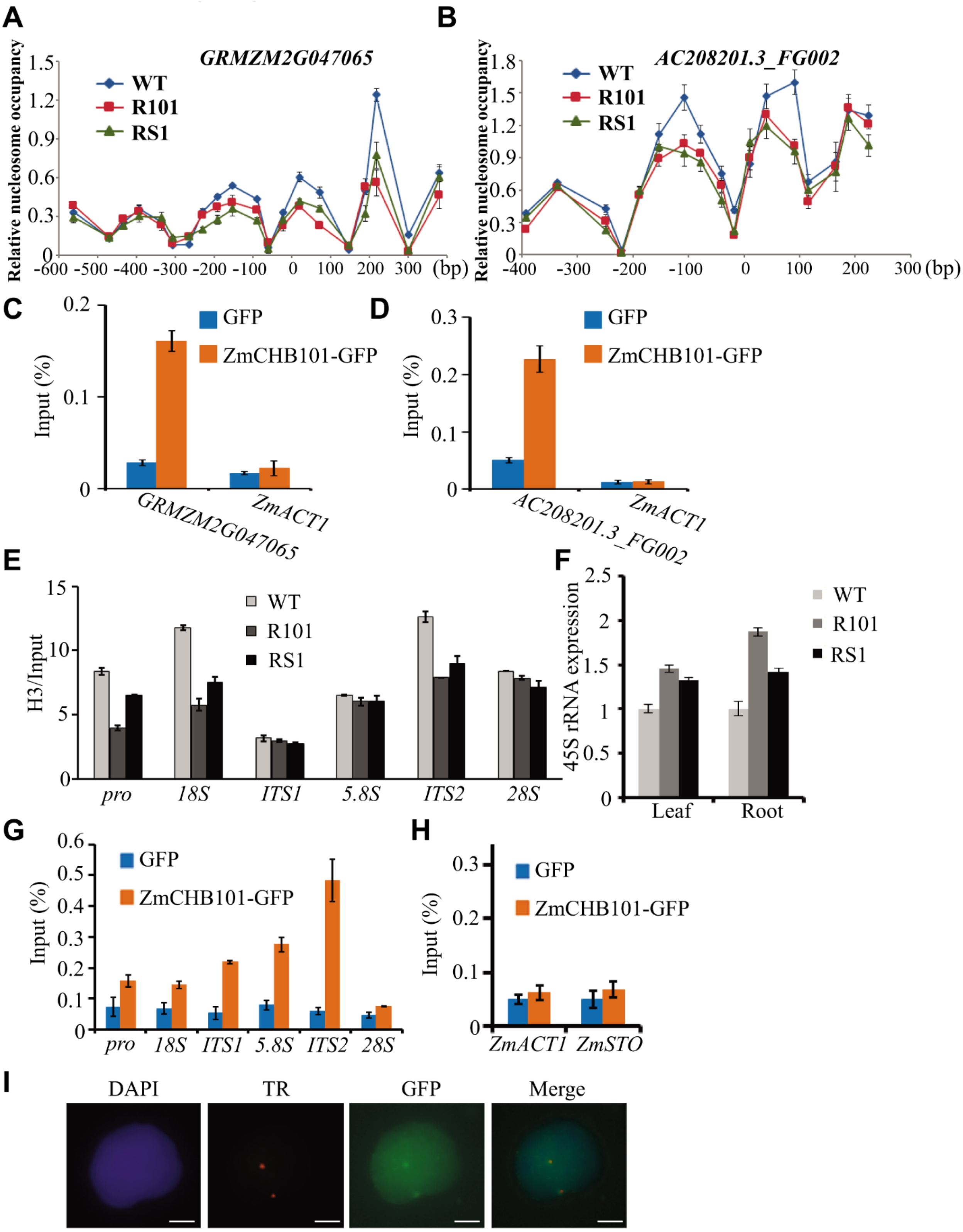

Fig. S5. **ZmCHB101 is required for maintaining nucleosome density and associates with *GRMZM2G047065, AC208201.3_FG002* and 45S rDNA.** (**A-B**) ZmCHB101 is required to maintain high occupancy of the nucleosomes at the upstream and gene body sites at *GRMZM2G047065* (**A**) and *AC208201.3_FG002* (**B**) loci. H3 Chip-qPCR were performed to monitor nucleosome positioning and occupancy at *GRMZM2G047065* (**A**)

and *AC208201.3_FG002* (**B**). 7-day-old wild-type (WT) , RS1 and R101 seedlings were used for nuclei extraction and DNA associated with histone H3 was immunoprecipitated with the anti-H3 antibody. X-axis denotes distance from the transcription start site and Y-axis denotes relative nucleosome occupancy. Error bars indicate SD (n=3). (**C-D**) ZmCHB101 could directly associate with UTS region of *GRMZM2G047065* (**C**) and *AC208201.3_FG002* (**D**). Protoplasts were transfected with *ZmCHB101-GFP* or *GFP* and nuclei were extracted for ChIP-qPCR. The y-axis values were the relative quantities of DNA. Error bars indicate SD (n=3). (**E**) Chip-qPCR analysis showed that the total level of histone H3 within 45S rDNA regions was decreased in ZmCHB101 RNAi lines. Primers specific for different region of 45S rDNAs were used to amplify DNA for quantitative real-time PCR. The y-axis values were the relative quantities of DNA and the x-axis indicated different regions of 45S rDNAs. Error bars indicate SD (n=3). (**F**) Expression of 45S rDNA was increased in RS1 and R101 compared with WT. *ZmUBI* was used as internal control. Each experiment was repeated three times and the average value was shown with the SD. (**G**) ZmCHB101 associates with 45S rDNA. Protoplasts transfected with *ZmCHB101-GFP* or *GFP* were used for ChIP-qPCR analysis. Error bars indicate SD (n=3). (**H**) *ZmACT1* and Stonor retrotransposon were used as negative controls. Error bars indicate SD (n=3). (**I**) ZmCHB101 colocalizes to 45S rDNA region. DAPI, nuclei were stained with DAPI for DNA; TR, 45S rDNA probes were lable with Texas Red-5-dCTP (red fluorescence); GFP, ZmCHB101-GFP fusion protein (green fluorescence). Bar=10 μm.
